# Molecular profiling of pediatric medulloblastoma in Kazakhstan: Genomic alterations, subgroup distribution, and survival

**DOI:** 10.1111/bpa.70144

**Published:** 2026-09-08

**Authors:** Aidos Bolatov, Askhat Zhakupov, Aizhan Abdikadirova, Malika Sapargaliyeva, Bakytkali Ibraimov, Zhanat Zhalimbetova, Gabit Ólenbay, Berik Zhetpisbayev, Xingzhi Xu, Mirgul Bayanova

**Affiliations:** ^1^ Guangdong Key Laboratory for Genome Stability and Disease Prevention and Carson International Cancer Center, Marshall Laboratory of Biomedical Engineering, Shenzhen University Medical School Shenzhen University Shenzhen China; ^2^ Clinical Academic Department of Laboratory Medicine, Pathology, and Genetics “University Medical Center” Corporate Fund Astana Kazakhstan; ^3^ Department of Pediatric Neurosurgery National Center for Neurosurgery Astana Kazakhstan; ^4^ Pathology Department National Center for Neurosurgery Astana Kazakhstan

**Keywords:** Kazakhstan, medulloblastoma, molecular profiling, pediatric brain tumor, whole‐exome sequencing

## Abstract

Medulloblastoma is the most common malignant pediatric brain tumor and comprises biologically distinct molecular subgroups with different clinicopathologic and prognostic characteristics. Molecular data from Kazakhstan and other underrepresented regions remain limited, and practical approaches for molecular subgroup assignment using formalin‐fixed, paraffin‐embedded (FFPE) material are needed in settings where advanced molecular classification is not routinely available. We retrospectively analyzed 40 pediatric medulloblastomas diagnosed between 2015 and 2024 at the Corporate Fund “University Medical Center,” Kazakhstan. Archived FFPE tumor material underwent histologic review, immunohistochemical evaluation (β‐catenin, YAP1, and GAB1), and whole‐exome sequencing. Tumors were assigned to WNT, SHH, or non‐WNT/non‐SHH categories using a combined morphologic, immunophenotypic, and genomic framework, and clinicopathologic variables and overall survival were evaluated across subgroups. WNT medulloblastomas (*n* = 7, 17.5%) showed the most canonical profile, characterized by classic histology, uniform β‐catenin nuclear positivity, recurrent *CTNNB1*/*APC* alterations, and frequent chromosome 6 loss. SHH medulloblastomas (*n* = 10, 25.0%) were enriched for desmoplastic/nodular morphology, frequent YAP1/GAB1 expression, pathogenic *PTCH1*/*SUFU* alterations, and additional events involving *TP53*, *TERT*, and focal amplifications in a subset. Non‐WNT/non‐SHH medulloblastomas (*n* = 23, 57.5%) showed the greatest genomic heterogeneity, including frequent i17q and broader structural complexity. Clinically, WNT tumors occurred predominantly in older children and had the most favorable survival, whereas non‐WNT/non‐SHH tumors were the only subgroup associated with metastatic disease at presentation and showed the poorest long‐term survival. Overall, pediatric medulloblastoma in this cohort demonstrated subgroup‐specific patterns consistent with established biology. The integration of pathology, immunohistochemistry, and sequencing enabled clinically meaningful molecular stratification. These findings expand evidence from an underrepresented setting and support pragmatic, resource‐adapted profiling in routine practice.

## Graphical abstract

A practical formalin‐fixed, paraffin‐embedded (FFPE)‐based molecular workflow integrating pathology, immunohistochemistry, and genomic profiling provides clinically meaningful subgroup classification of pediatric medulloblastoma and expands molecular evidence from an underrepresented Central Asian population.

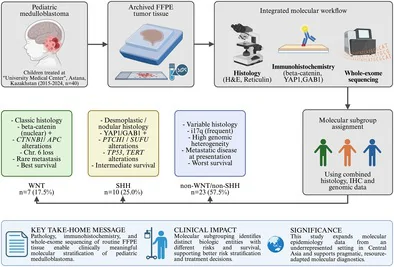

## 1 INTRODUCTION

Medulloblastoma is the most common malignant brain tumor in children and one of the most clinically significant embryonal tumors of the central nervous system [1, 2]. It accounts for approximately 20% of all pediatric brain tumors and typically arises in the posterior fossa during the first decade of life [1, 3, 4, 5]. The annual incidence is estimated at 2–5.8 cases per million children, with a consistent male predominance reported across populations [6, 7, 8, 9].

Despite substantial advances in multimodal treatment, medulloblastoma remains an aggressive pediatric malignancy characterized by a high propensity for cerebrospinal fluid dissemination and disease recurrence [8, 10]. Contemporary management combines maximal safe surgical resection, craniospinal irradiation, and multi‐agent chemotherapy, resulting in markedly improved survival in high‐resource settings [9, 10]. However, these therapeutic gains have been accompanied by considerable long‐term treatment‐related morbidity, with survivors frequently experiencing neurocognitive impairment, endocrine dysfunction, chronic medical complications, reduced functional independence, and impaired quality of life [8, 11, 12].

The classification of medulloblastoma has evolved from a predominantly histology‐based framework to a molecularly informed diagnostic model. According to the current World Health Organization (WHO) classification of central nervous system tumors, medulloblastoma comprises WNT‐activated, Sonic hedgehog (SHH)‐activated and TP53‐wildtype, SHH‐activated and TP53‐mutant, and non‐WNT/non‐SHH tumors, the latter broadly encompassing neoplasms historically corresponding to Group 3 and Group 4 lineages [5, 10, 13, 14, 15, 16, 17]. These molecular categories differ substantially in age distribution, driver alterations, copy‐number profiles, metastatic behavior, and clinical outcome, and have become central to contemporary risk stratification, clinical trial design, and treatment de‐escalation or intensification strategies [18, 19, 20, 21, 22].

Despite these advances, implementation of molecular subgrouping remains challenging in many centers. DNA methylation profiling is considered the reference standard for molecular classification but is frequently limited by cost, infrastructure, bioinformatic requirements, and tissue‐quality constraints, particularly in retrospective formalin‐fixed, paraffin‐embedded (FFPE) cohorts [17, 23, 24, 25]. Immunohistochemical surrogate panels based on β‐catenin, yes‐associated protein 1 (YAP1), and GRB2‐associated binding protein 1 (GAB1) provide a practical alternative for distinguishing WNT and SHH tumors from non‐WNT/non‐SHH tumors; however, they cannot reliably separate Group 3 from Group 4 [5, 26]. Consequently, integrated diagnostic strategies combining histology, immunohistochemistry, and sequencing‐based genomic analyses remain highly relevant for resource‐limited settings, where clinically meaningful molecular stratification may still be achieved using routinely available FFPE material [15, 27, 28, 29, 30, 31].

Published molecular data on pediatric medulloblastoma in Kazakhstan remain extremely limited. Previous studies have largely been restricted to targeted analysis of *TP53* and *CTNNB1* or descriptive clinicopathologic reports without comprehensive molecular subgrouping [32, 33]. To our knowledge, Kazakhstan has not previously reported an integrated analysis combining histopathology, immunohistochemistry, whole‐exome sequencing (WES), and copy‐number analysis for molecular characterization of pediatric medulloblastoma. This knowledge gap reflects the broader scarcity of molecular neuro‐oncology data across Central Asia [34].

In this context, the present study aimed to characterize the molecular profile of pediatric medulloblastoma in Kazakhstan using an FFPE‐applicable retrospective framework integrating histopathology, immunohistochemistry, WES, and copy‐number analysis. Because WES does not reliably distinguish Group 3 from Group 4 in the absence of DNA methylation profiling or transcriptomic classification, tumors lacking canonical WNT or SHH features were intentionally retained within the WHO‐recognized non‐WNT/non‐SHH category. Specifically, we sought to: (1) assign tumors to WNT, SHH, or non‐WNT/non‐SHH categories using integrated morphologic, immunophenotypic, and genomic features; (2) characterize subgroup‐associated sequence variants and copy‐number alterations (CNAs); (3) examine the clinicopathologic distribution of these molecular categories; and (4) explore their relationship with survival outcomes in a Kazakhstani pediatric cohort.

## 2 MATERIALS AND METHODS

### 2.1 Study design and patients

This retrospective cohort study included pediatric patients with medulloblastoma who were treated between 2015 and 2024 at the Corporate Fund “University Medical Center,” Astana, Kazakhstan. During the study period, 168 pediatric patients with medulloblastoma were managed at our institution. Archived FFPE tumor tissue was available for 45 cases, which were independently reviewed by two experienced neuropathologists (Corporate Fund “University Medical Center” and the National Center for Neurosurgery, Astana, Kazakhstan) to confirm the diagnosis according to the applicable WHO classification criteria. Following histopathologic review, two cases were reclassified as alternative histopathologic entities and were therefore excluded from further analyses. Among the remaining cases, three were excluded because the available FFPE tissue was insufficient for molecular testing or did not meet pre‐analytical quality control requirements for DNA extraction and WES. Consequently, the final study cohort consisted of 40 histologically confirmed pediatric medulloblastoma cases. Archived FFPE tumor material from these cases was subsequently retrieved for histopathologic reassessment, immunohistochemical analysis, and molecular profiling.

### 2.2 Clinical data collection

Clinical and demographic data were extracted retrospectively from available medical records. Variables collected included sex, age at diagnosis, tumor stage, metastatic status, extent of resection, postoperative magnetic resonance imaging (MRI) residual tumor status, and vital status at last follow‐up. Age was analyzed both as a continuous variable and by predefined categories. Tumor stage was recorded using the local clinical staging system applied in the cohort (T1–T4) and metastatic status, where available. Survival time was calculated from the date of diagnosis to the date of death or last available follow‐up.

All patients were managed according to the national pediatric medulloblastoma treatment protocol of the Republic of Kazakhstan, which is derived from the HIT 2000 and HIT‐REZ 2005 therapeutic strategies. Management followed a standardized multimodal approach incorporating neurosurgical resection, radiotherapy, and systemic chemotherapy, with treatment intensity adapted to the individual patient's risk profile. Following maximal safe tumor resection, postoperative MRI performed within 24–48 h was used to evaluate residual disease, and together with patient age, histologic subtype, metastatic dissemination (Chang M stage), and postoperative residual tumor status (Chang R stage), informed risk stratification and subsequent treatment planning. Children aged 4 years or older generally received craniospinal irradiation at 23.4 Gy for standard‐risk disease or 36 Gy for high‐risk disease, followed by irradiation of the primary tumor bed to a cumulative dose of 54 Gy. For younger children, radiotherapy was deferred or modified in accordance with the national protocol to reduce treatment‐related toxicity. Systemic chemotherapy was administered using age‐ and risk‐adapted HIT‐based regimens. Although the specific therapeutic intensity varied according to predefined clinical risk factors, all patients were treated within a uniform national protocol throughout the study period, thereby ensuring a consistent therapeutic framework for outcome assessment.

### 2.3 Histology and immunohistochemistry

Archived tumor material was reviewed on hematoxylin and eosin (H&E)‐stained sections prepared from FFPE tissue blocks. Serial sections of 3–4 μm thickness were cut and examined according to standard histopathologic procedures. Histologic evaluation included assessment of tissue architecture, cellular composition, cytomorphologic features, degree of differentiation, and mitotic activity. Histologic subtype was categorized as classic, desmoplastic/nodular (D/N), medulloblastoma with extensive nodularity (MBEN), or large cell/anaplastic (LCA) according to standard neuropathologic criteria.

Histochemical analysis was performed using silver impregnation staining in accordance with a validated laboratory protocol. This method was used to visualize reticulin fibers and to assess stromal‐parenchymal relationships, particularly in tumors with nodular or desmoplastic features.

Immunohistochemical staining was performed using an automated Leica Bond‐III Max immunostainer (Leica Biosystems). Primary antibodies included β‐catenin (Leica Biosystems), as well as GAB1 and YAP1 (Thermo Fisher Scientific). Before the main analysis, immunohistochemical protocols for all antibodies were validated and optimized, including selection of the appropriate primary antibody titer, antigen retrieval conditions, and assessment of staining specificity. Positive controls consisted of tissues with known expression of the corresponding markers, while negative controls were generated by omission of the primary antibody.

Immunohistochemical staining was independently evaluated by two experienced pathologists. Marker interpretation was based on the characteristic staining pattern and subcellular localization described in established medulloblastoma subgrouping studies rather than on predefined percentage cutoffs. Immunohistochemical expression was evaluated semiquantitatively with attention to subcellular localization (nuclear, cytoplasmic, and membranous), staining intensity, and the proportion of positive tumor cells. β‐catenin positivity was defined by the presence of unequivocal nuclear staining in tumor cells, irrespective of the proportion of positive cells, consistent with established medulloblastoma diagnostic recommendations. Tumors showing exclusively membranous and/or cytoplasmic staining were considered negative for WNT‐pathway activation. Because focal nuclear β‐catenin accumulation may occur in WNT‐activated medulloblastomas, interpretation relied on the presence of unequivocal nuclear localization rather than predefined percentage or intensity thresholds. YAP1 positivity was defined by distinct nuclear immunoreactivity in tumor cells, with endothelial‐cell staining serving as an internal positive control. GAB1 positivity was defined by cytoplasmic immunoreactivity in tumor cells and was interpreted as an additional supportive finding for SHH activation rather than a mandatory criterion for subgroup assignment [5, 35, 36]. All immunohistochemical findings were interpreted in conjunction with histologic features and genomic data within the integrated molecular classification framework.

Histopathologic review and diagnostic confirmation were completed before molecular analyses were performed. Accordingly, the reviewing pathologists were blinded to the WES and copy‐number analysis results during morphologic evaluation. In selected diagnostically challenging cases, an independent expert pathology consultation was obtained from an experienced pathologist at Nagasaki University (Nagasaki, Japan), and these reviews were likewise performed before molecular testing.

### 2.4 DNA extraction and whole‐exome sequencing

Genomic DNA was extracted from archived FFPE tumor sections using the ReliaPrep FFPE gDNA Miniprep System (Promega, Madison, WI, USA) according to the manufacturer's instructions. Briefly, sections were deparaffinized using mineral oil (300–500 μL, depending on section thickness), followed by incubation at 80°C for 1 min with mixing. Tissue lysis was performed using 200 μL lysis buffer and 20 μL proteinase K at 56°C for 1 h, followed by 80°C for 4 h to reverse formalin‐induced cross‐links. After cooling, RNA was removed by treatment with 10 μL RNase A at room temperature for 5 min. DNA was purified using spin columns and eluted in 30–50 μL of elution buffer.

DNA concentration and purity were initially assessed using a NanoDrop spectrophotometer (Thermo Fisher Scientific, Waltham, MA, USA), including A260/A280 ratio measurement. DNA fragmentation and integrity were further evaluated by 1.5% agarose gel electrophoresis in 1× Tris‐acetate‐EDTA buffer, with imaging performed using the I‐BRIGHT GelDoc system (Thermo Fisher Scientific). Fluorometric quantification was performed using the QuantiFluor dsDNA System (Promega) on a Victor Nivo Multimode Microplate Reader (PerkinElmer, Waltham, MA, USA). DNA integrity number (DIN) was assessed using the Agilent TapeStation 4200 (Agilent Technologies, Santa Clara, CA, USA).

DNA concentration ranged from 2.2 to 331 ng/μL, with total DNA yields between 0.05 and 8.5 μg. Most samples showed DIN values between 1.0 and 3.2, consistent with FFPE‐derived DNA. All included samples passed pre‐analytical quality assessment and were considered suitable for downstream library preparation and sequencing.

WES was performed by Macrogen Inc. (Seoul, Republic of Korea). Library preparation and exome capture were conducted using the Twist Human Core Exome 2.0 (FFPE) kit (Twist Bioscience, South San Francisco, CA, USA) according to the manufacturer's protocol. A total of 50 ng genomic DNA was used for library construction. DNA was enzymatically fragmented to a peak size of approximately 200 bp. Libraries were indexed with TruSeq‐compatible adapters, amplified, and subjected to quality control using the Agilent TapeStation and quantitative polymerase chain reaction with the KAPA Library Quantification Kit. Hybridization and target enrichment were performed according to the Twist Bioscience protocol. Sequencing was carried out on an Illumina platform in paired‐end mode (2 × 151 bp).

### 2.5 Bioinformatics, variant interpretation, and orthogonal validation

Primary basecalling and demultiplexing were performed using bcl‐convert. Raw read quality was assessed with FastQC. Reads were aligned to the GRCh38/hg38 human reference genome (National Center for Biotechnology Information, February 2022 release) using BWA‐MEM v0.7.17. Duplicate reads were marked and removed using Picard v3.1.1. Base quality score recalibration and variant calling were performed using GATK v4.5.0.0. Variant annotation was conducted using SnpEff v5.2, with reference to the database of single nucleotide polymorphisms build 156, 1000 Genomes Phase 3, ClinVar (July 2024 release), and ESP6500.

Variant interpretation was performed using the Franklin by QIAGEN platform, which applies American College of Medical Genetics and Genomics/Association for Molecular Pathology (ACMG/AMP)‐aligned criteria for sequence variant assessment. Variants were classified according to the ACMG/AMP framework as pathogenic, likely pathogenic, variant of uncertain significance (VUS), likely benign, or benign [37]. Oncogenicity interpretation was additionally informed by the ClinGen/CGC/VICC somatic oncogenicity recommendations [38]. For cancer‐related clinical significance, somatic variants were also categorized according to the AMP/ASCO/CAP four‐tier system, in which Tier I denotes variants of strong clinical significance, Tier II variants of potential clinical significance, Tier III variants of unknown clinical significance, and Tier IV benign or likely benign variants [39].

Variant interpretation incorporated multiple lines of evidence, including population allele frequency, predicted functional impact, prior database annotation, and published evidence where available. Curated resources included ClinVar, the Catalogue of Somatic Mutations in Cancer, and the Genome Aggregation Database.

Selected single‐nucleotide variants (SNVs) were validated by Sanger sequencing. In addition, selected CNAs were assessed by multiplex ligation‐dependent probe amplification (MLPA) using SALSA MLPA probe mixes P301 and P302 (MRC‐Holland), covering chromosomal regions on 2, 3, 6, 7, 9, 14q, 16, and 17. Reactions were performed according to the manufacturer's protocol, fragments were analyzed on an Applied Biosystems 3500 Genetic Analyzer, and data were processed in Coffalyser.Net using population‐based normalization. Copy‐number ratios of 0.8–1.2 were considered consistent with a normal diploid state. MLPA was used as an orthogonal supportive method for selected WES‐derived CNA findings.

### 2.6 Molecular classification strategy

Molecular classification was performed within the current WHO framework for medulloblastoma, which recognizes medulloblastoma as a molecularly defined entity comprising WNT‐activated, SHH‐activated, and non‐WNT/non‐SHH categories. Because DNA methylation profiling and transcriptome‐based classification were not available in this retrospective FFPE cohort, subgroup assignment was based on an integrated assessment of histology, immunohistochemistry, and WES/copy‐number data, using established subgroup‐associated surrogate markers and genomic correlates described in neuropathology and medulloblastoma classification studies [1, 2, 3, 4, 5, 6, 7, 17, 40].

**FIGURE 1:**
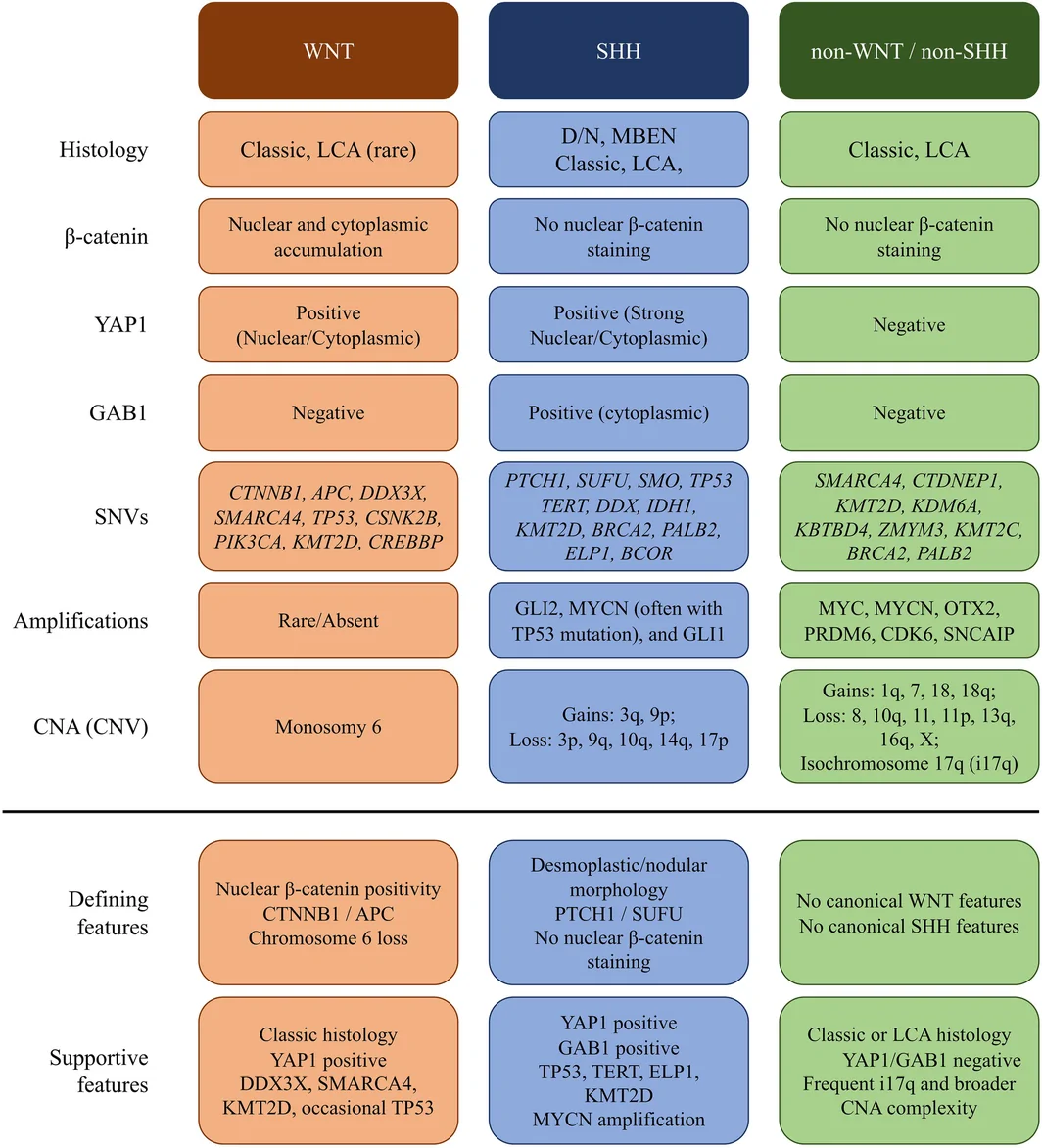
Study‐specific framework for integrated molecular classification of pediatric medulloblastoma. The scheme summarizes the operational criteria used for assignment of tumors to the WNT, SHH, or non‐WNT/non‐SHH categories in this study. Classification was based on combined evaluation of histology, immunohistochemistry, and genomic findings. Because DNA methylation profiling was not available, non‐WNT/non‐SHH tumors were not further subdivided into Group 3 and Group 4. CNA, copy‐number alteration; D/N, desmoplastic/nodular; LCA, large cell/anaplastic; SNVs, single‐nucleotide variant.

As summarized in Figure 1, subgroup assignment followed a study‐specific, literature‐informed interpretive framework. Tumors were assigned to the WNT subgroup when they demonstrated evidence of WNT‐pathway activation, defined primarily by nuclear β‐catenin immunoreactivity and/or canonical WNT‐pathway alterations such as pathogenic variants in *CTNNB1* or *APC*. Additional supportive findings included classic histology, YAP1 positivity, absence of GAB1 expression, recurrent co‐alterations such as *DDX3X*, *SMARCA4*, or *KMT2D*, and chromosome 6 loss where present. This approach is consistent with prior studies showing that WNT‐activated medulloblastomas typically demonstrate nuclear β‐catenin accumulation, frequent *CTNNB1* alteration, and monosomy 6 [5, 40, 41].

Tumors were assigned to the SHH subgroup when they showed a profile compatible with SHH‐pathway activation, including desmoplastic/nodular morphology and/or an immunophenotype characterized by YAP1 expression. GAB1 expression, when present, was interpreted as an additional supportive finding rather than a mandatory criterion for subgroup assignment, provided that nuclear β‐catenin staining was absent. Pathogenic alterations in SHH‐pathway genes, most notably *PTCH1* or *SUFU*, were considered primary genomic evidence of SHH‐pathway activation when identified. By contrast, alterations involving *TP53*, *TERT* promoter, *ELP1*, *KMT2D*, and focal *MYCN* amplification were interpreted as secondary or cooperative genomic events. Although these alterations are characteristic of subsets of SHH medulloblastoma and contribute to biological heterogeneity and risk stratification, they do not constitute direct evidence of SHH‐pathway activation and therefore were not used as primary determinants of subgroup assignment. Instead, they served as supportive molecular findings once SHH activation had been established through integrated histopathologic, immunophenotypic, and genomic evaluation. Although *TP53* alterations were evaluated by WES, they were interpreted within this framework as secondary genomic events and did not influence primary subgroup assignment. Accordingly, SHH tumors were assigned based on canonical evidence of SHH‐pathway activation rather than *TP53* status. Furthermore, because standardized p53 immunohistochemistry was not available for the entire retrospective cohort and the primary objective of this study was to establish the major molecular subgroups (WNT, SHH, and non‐WNT/non‐SHH) using an integrated FFPE‐applicable workflow, SHH tumors were not further subclassified as *TP53*‐mutant or *TP53*‐wildtype according to the current WHO classification. This strategy is aligned with validated FFPE‐applicable immunohistochemical panels and neuropathology reviews indicating that β‐catenin, YAP1, and GAB1 are useful surrogate markers for medulloblastoma subgrouping, with *PTCH1*/*SUFU* alterations representing canonical SHH‐pathway lesions [5, 40].

Tumors lacking canonical features of either WNT or SHH activation were classified as non‐WNT/non‐SHH. Operationally, this category was defined by the absence of subgroup‐defining WNT and SHH signatures based on integrated histologic, immunohistochemical, and genomic assessment. Supportive features included classic or LCA histology, absence of nuclear β‐catenin staining, generally absent YAP1 and GAB1 expression, and a broader genomic profile characterized by heterogeneous sequence variants and structurally complex CNAs, including frequent i17q. Because the study design did not include DNA methylation profiling or transcriptome‐based classification, reliable discrimination between Group 3 and Group 4 was not feasible. Although WES provides valuable information on sequence variants and CNAs, it does not capture the epigenetic signatures required to distinguish these closely related molecular entities. Therefore, tumors lacking canonical WNT or SHH features were intentionally retained within the broader WHO‐recognized non‐WNT/non‐SHH category rather than assigned to a more specific molecular subclass. This approach is consistent with current evidence demonstrating that immunohistochemistry can reliably distinguish WNT‐activated and SHH‐activated medulloblastomas from non‐WNT/non‐SHH tumors, whereas accurate separation of Group 3 and Group 4 generally requires DNA methylation profiling or transcriptomic approaches [4, 17, 26]. Final subgroup assignment was based on the overall integrated profile rather than on any single marker in isolation. In cases with partial discordance between morphology, immunophenotype, and genomic findings, greater weight was given to canonical pathway‐defining features, while tumors lacking such defining features were conservatively retained within the non‐WNT/non‐SHH category to avoid overinterpretation of the available molecular data. The complete study‐specific classification framework is illustrated in Figure 1.

### 2.7 Statistical analysis

Descriptive statistics were used to summarize demographic, clinical, histologic, immunohistochemical, and molecular characteristics. Categorical variables were presented as counts and percentages, and continuous variables as median and range. Comparisons of categorical variables across molecular subgroups were performed using the chi‐square test or Fisher's exact test, as appropriate. A two‐sided *p* value <0.05 was considered statistically significant.

Overall survival (OS) was defined as the time from diagnosis to death from any cause or last follow‐up. Survival probabilities were estimated using the Kaplan–Meier method, and subgroup differences were assessed primarily using the log‐rank test. Additional weighted comparisons were explored using Gehan, Tarone‐Ware, and Peto‐Peto tests. Median OS and estimated 3‐ and 5‐year OS rates were calculated for the overall cohort and for each molecular subgroup.

### 2.8 Ethical issues

This study was approved by the Local Committee of Bioethics of the Corporate Fund “University Medical Center,” Kazakhstan (Protocol No. 8/2024/ПЭ, dated September 03, 2024). Written informed consent for participation and for the use of clinical records and biomaterial in anonymized research was obtained from the legal representatives of patients whenever feasible during the study period. For archived cases, the original hospitalization consent documentation included permission for the use of anonymized clinical data and biological material for research purposes. All study procedures were carried out in accordance with the principles of the Declaration of Helsinki and the applicable ethical and regulatory requirements, including internal normative documents of the Republic of Kazakhstan.

## 3 RESULTS

### 3.1 Cohort characteristics

The study cohort comprised 40 pediatric patients with medulloblastoma. The median age at diagnosis was 8.0 years (range, 0.5–16.0 years), and the majority of patients were aged 3–10 years (21/40, 52.5%). A clear male predominance was observed, with 28/40 patients (70.0%) being male. The median follow‐up/time to outcome was 63.0 months (range, 12–214 months).

Clinicopathologic characteristics stratified by integrated molecular subgroup are summarized in Table 1. Data on tumor stage, metastatic status, surgical resection category, and postoperative MRI residual tumor status were available for 39 patients, and percentages for these variables were calculated using the number of evaluable cases as the denominator. Most tumors presented with advanced local extension, with T3b being the most frequent stage (13/39, 33.3%), followed by T2 (11/39, 28.2%) and T3a (9/39, 23.1%). Less frequent categories included T4 (4/39, 10.3%) and T1 (2/39, 5.1%). Metastatic disease at diagnosis was documented in 8/39 patients (20.5%).

**TABLE 1:** Clinicopathologic characteristics of the cohort according to integrated molecular subgroup (*N* = 40).

Variable (*n*, %)	By molecular subgroup, *n* (%)			
	WNT	SHH	Non‐WNT/non‐SHH	χ^2^, *p*
Sex				
Male (28, 70.0%)	4 (57.1)	5 (50.0)	19 (82.6)	4.20, *p* = 0.123
Female (12, 30.0%)	3 (42.9)	5 (50.0)	4 (17.4)	
Age group				
<3 years old (6, 15.0%)	‐	2 (20.0)	4 (17.4)	13.8, *p* = 0.008
3–10 years old (21, 52.5%)	1 (14.3)	4 (40.0)	16 (69.6)	
>10 years old (13, 32.5%)	6 (85.7)	4 (40.0)	3 (13.0)	
Tumor stage, *n* = 39				
T1 (2, 5.1%)	‐	1 (10.0)	1 (4.5)	7.22, *p* = 0.513
T2 (11, 28.2%)	3 (42.9)	4 (40.0)	4 (18.2)	
T3a (9, 23.1%)	2 (28.6)	3 (30.0)	4 (18.2)	
T3b (13, 33.3%)	2 (28.6)	2 (20.0)	9 (40.9)	
T4 (4, 10.3%)	‐	‐	4 (18.2)	
Metastasis, *n* = 39				
No (31, 79.5%)	7 (100)	10 (100)	14 (63.6)	7.78, *p* = 0.020
Yes (8, 20.5%)	‐	‐	8 (36.4)	
Surgical resection grade (S), *n* = 39				
S1 (9, 23.1%)	4 (57.1)	3 (30.0)	2 (9.1)	9.67, *p* = 0.139
S2 (14, 35.9%)	1 (14.3)	3 (30.0)	10 (45.5)	
S3 (13, 33.3%)	2 (28.6)	4 (40.0)	7 (31.8)	
S4 (3, 7.7%)	‐	‐	3 (13.6)	
Postoperative MRI residual tumor (R) status, *n* = 39				
R1 (9, 23.1%)	4 (57.1)	3 (30.0)	2 (9.1)	11.2, *p* = 0.082
R2 (10, 25.6%)	1 (14.3)	1 (10.0)	8 (36.4)	
R3 (17, 43.6%)	2 (28.6)	6 (60.0)	9 (40.9)	
R4 (3, 7.7%)	‐	‐	3 (13.6)	
Vital status at last follow‐up				
Alive (26, 65.0%)	7 (100)	7 (70.0)	12 (52.2)	5.54, *p* = 0.063
Dead (14, 35.0%)	‐	3 (30.0)	11 (47.8)	

*Note*: T1, localized disease; T2, tumor ≥3 cm invading adjacent structures or partially filling the fourth ventricle; T3a, extension into the aqueduct or foramina of Magendie/Luschka with hydrocephalus; T3b, tumor filling the fourth ventricle with brainstem infiltration; T4, extension into the midbrain, third ventricle, or upper spinal cord. S1, gross total resection; S2, subtotal resection; S3, partial resection (>1.5 cm residual); S4, biopsy only. R1, MRI‐confirmed absence of residual tumor; R2, marginal contrast enhancement; R3, visible residual tumor; R4, no significant postoperative change.

Surgical data were also available for 39 cases. Gross total resection (S1) was achieved in 14/39 patients (35.9%), while subtotal resection (S2) was performed in 13/39 (33.3%). Partial resection (S3) and biopsy‐only procedures (S4) accounted for 9/39 (23.1%) and 3/39 (7.7%) cases, respectively. On postoperative MRI, visible residual tumor (R3) was the most frequent finding, identified in 17/39 patients (43.6%), whereas MRI‐confirmed absence of residual tumor (R1) was observed in 9/39 (23.1%), marginal enhancement (R2) in 10/39 (25.6%), and no significant postoperative change (R4) in 3/39 (7.7%).

At the last available follow‐up, 26/40 patients (65.0%) were alive and 14/40 (35.0%) had died. Overall, the cohort was characterized by male predominance, a predominance of patients in the 3‐ to 10‐year age group, and a substantial proportion of tumors presenting with intermediate‐to‐advanced local stage, with approximately one‐fifth of evaluable patients showing metastatic disease at diagnosis.

### 3.2 Integrated molecular classification of the cohort

**FIGURE 2:**
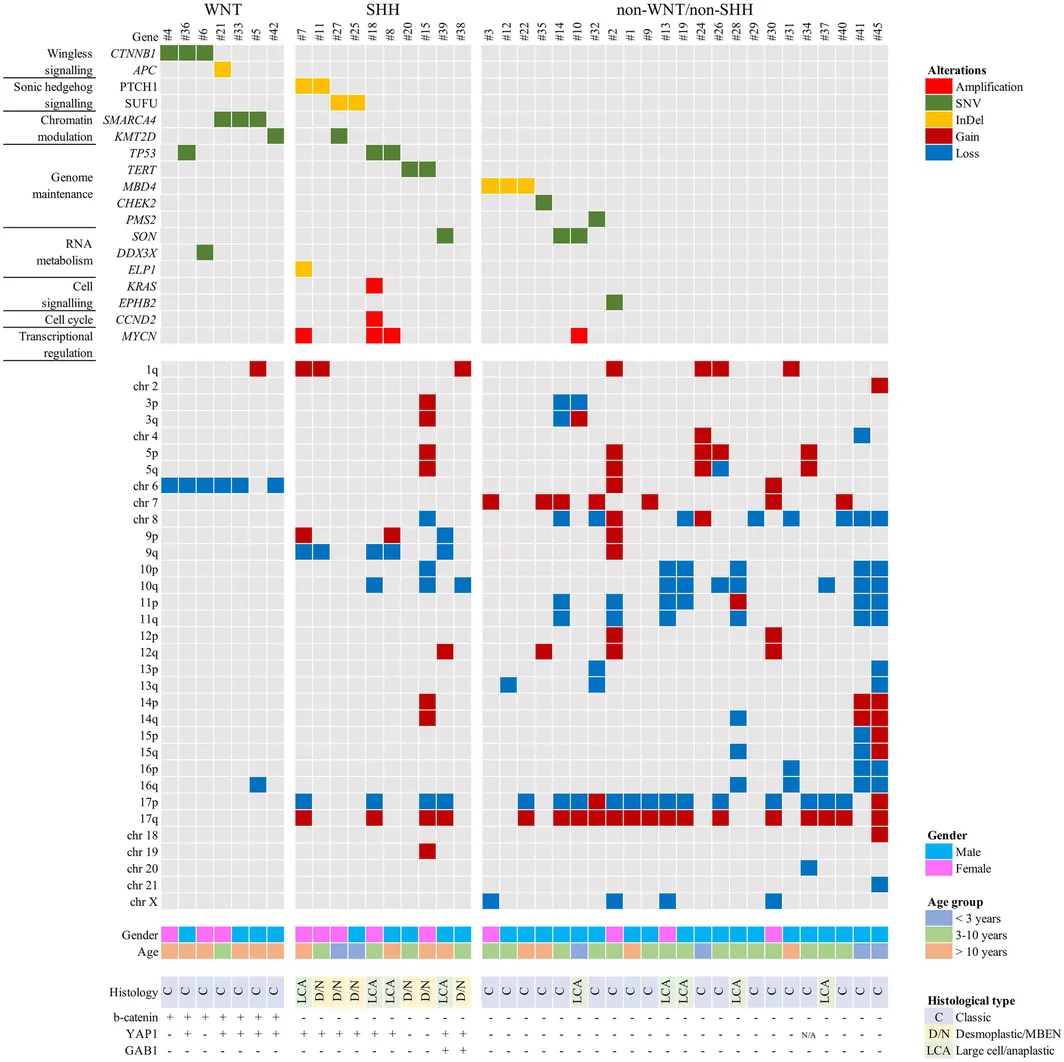
Integrated clinicopathologic and genomic landscape of pediatric medulloblastoma according to molecular subgroup. The upper panel shows clinically and biologically relevant genetic alterations, organized by functional category. The middle panel summarizes recurrent copy‐number alterations across chromosomal arms and whole chromosomes. The lower annotation tracks indicate sex, age group, histologic subtype, and immunohistochemical findings (β‐catenin, YAP1, and GAB1). C, classic; D/N, desmoplastic/nodular; InDel, insertion/deletion; LCA, large cell/anaplastic; MBEN, medulloblastoma with extensive nodularity; N/A, NA, not available; SNV, single‐nucleotide variant. Columns represent individual tumors. Molecular subgroup assignment was based on integrated histologic, immunohistochemical, and genomic evaluation. “+” indicates positive staining and “−” indicates negative staining.

Using the predefined integrated classification framework, all 40 medulloblastomas were assigned to one of three molecular categories: WNT (7/40, 17.5%), SHH (10/40, 25.0%), and non‐WNT/non‐SHH (23/40, 57.5%) (Figure 2). The non‐WNT/non‐SHH subgroup represented the largest proportion of cases.

Subgroup assignment was supported by concordant histologic, immunophenotypic, and genomic features. WNT tumors showed the most canonical profile, combining uniform β‐catenin positivity, recurrent WNT‐pathway alterations, and frequent chromosome 6 loss. SHH tumors were enriched for desmoplastic/nodular histology, YAP1 expression, and pathogenic alterations in *PTCH1* or *SUFU*. In contrast, non‐WNT/non‐SHH tumors lacked canonical WNT or SHH features and displayed the broadest histologic and genomic heterogeneity, including frequent i17q and multiple chromosomal imbalances.

These findings established clear subgroup‐specific patterns and provided the framework for subsequent clinicopathologic, genomic, and survival analyses.

### 3.3 Histopathologic and immunohistochemical features according to subgroup

**FIGURE 3:**
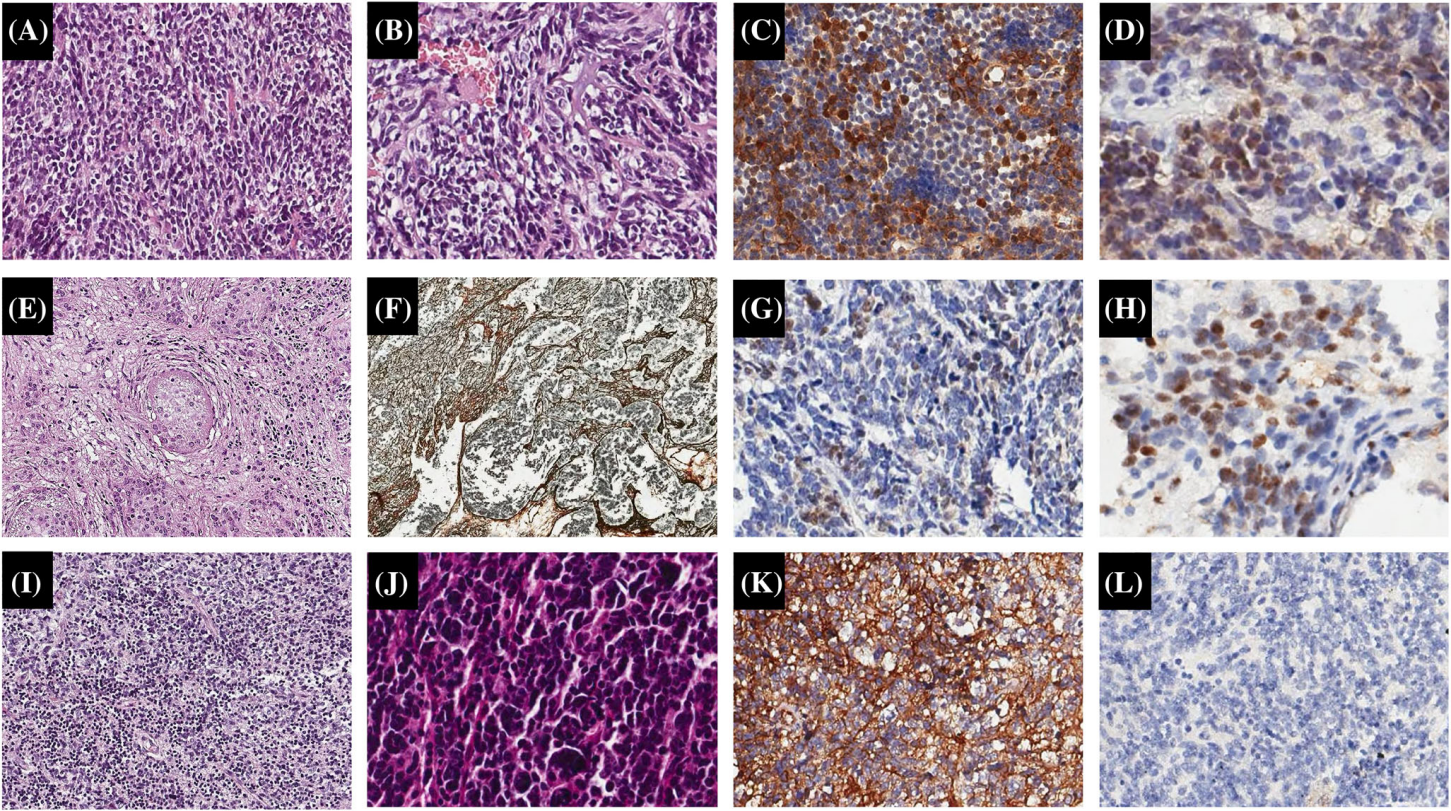
Representative hematoxylin and eosin (H&E), reticulin (silver), and immunohistochemical images illustrating the characteristic morphologic and immunophenotypic features of the three principal molecular subgroups of medulloblastoma. (A–D) WNT subgroup: (A) classic histology on H&E staining (low‐power view, 10×); (B) classic histology on H&E staining (high‐power view, 40×); (C) characteristic nuclear β‐catenin expression consistent with WNT‐pathway activation; and (D) positive nuclear YAP1 immunoreactivity. (E–H) SHH subgroup: (E) desmoplastic/nodular morphology on H&E staining; (F) reticulin (silver) staining highlighting the characteristic desmoplastic architecture; (G) positive nuclear YAP1 immunoreactivity; and (H) positive GAB1 immunohistochemical expression. (I–L) Non‐WNT/non‐SHH subgroup: (I) classic morphology on H&E staining (low‐power view, 10×); (J) large cell/anaplastic morphology on H&E staining (high‐power view, 40×); (K) absence of nuclear β‐catenin expression with preserved membranous staining; and (L) absence of YAP1 immunohistochemical expression.

Marked subgroup‐specific differences were observed in both histologic and immunohistochemical profiles. All WNT tumors (7/7) exhibited classic histology, with no desmoplastic/nodular or LCA cases identified in this subgroup. Immunohistochemically, all WNT tumors showed characteristic nuclear β‐catenin positivity, consistent with their integrated molecular assignment. YAP1 expression was present in 5/7 cases, whereas GAB1 was negative in all tumors. Representative histopathological and immunohistochemical features of the WNT subgroup, including classic morphology, nuclear β‐catenin accumulation, and YAP1 expression, are shown in Figure 3A–D.

The SHH subgroup displayed a distinct morphologic and immunophenotypic pattern. Desmoplastic/nodular histology was observed in 6/10 tumors (60.0%), while the remaining 4/10 (40.0%) were classified as LCA. YAP1 positivity was detected in 8/10 cases (80.0%), whereas GAB1 positivity was observed in only 2/10 tumors (20.0%). Thus, SHH tumors were enriched for desmoplastic/nodular morphology and YAP1 expression, although GAB1 was less consistently expressed in this cohort. Representative examples of the characteristic desmoplastic/nodular architecture demonstrated by H&E and reticulin (silver) staining together with YAP1 and GAB1 immunoreactivity are presented in Figure 3E–H.

The non‐WNT/non‐SHH subgroup was composed predominantly of classic medulloblastomas (18/23, 78.3%) with a smaller subset of LCA tumors (5/23, 21.7%). Immunohistochemically, non‐WNT/non‐SHH tumors lacked the canonical WNT‐ and SHH‐associated staining patterns, with absence of nuclear β‐catenin and generally absent YAP1 and GAB1 expression. Representative histologic and immunohistochemical findings for this subgroup, including classic and LCA morphology, membranous β‐catenin staining without nuclear accumulation, and absence of YAP1 expression, are shown in Figure 3I–L.

Taken together, these findings indicate that routine histology and immunohistochemistry provided strong support for integrated subgroup assignment, with the highest concordance observed in WNT and SHH tumors. Representative examples of these subgroup‐specific morphologic and immunophenotypic features are illustrated in Figure 3.

### 3.4 Clinicopathologic characteristics by molecular subgroup

The three integrated molecular subgroups showed distinct clinicopathologic distributions (Table 1). Age at diagnosis differed significantly across groups (*p* = 0.008). WNT tumors occurred predominantly in older children, with 6/7 cases (85.7%) diagnosed in patients >10 years of age, and no cases observed in children younger than 3 years. In contrast, SHH tumors showed a broader age distribution, including 2/10 cases (20.0%) in children <3 years, 4/10 (40.0%) in those aged 3–10 years, and 4/10 (40.0%) in patients >10 years. The non‐WNT/non‐SHH subgroup was concentrated mainly in the 3‐ to 10‐year age range (16/23, 69.6%), with fewer cases among the youngest and oldest patients.

Sex distribution did not differ significantly among subgroups (*p* = 0.123), although descriptive differences were observed. The non‐WNT/non‐SHH subgroup showed the strongest male predominance (19/23, 82.6%), whereas the WNT and SHH groups were more balanced (WNT: 4/7 males, 57.1%; SHH: 5/10 males, 50.0%).

Clear differences were also observed in disease burden at presentation. Metastatic disease at diagnosis was identified exclusively in the non‐WNT/non‐SHH subgroup (8/23 cases), whereas no metastatic tumors were observed in the WNT or SHH groups (*p* = 0.020). Local tumor stage also showed variation across subgroups, although this did not reach statistical significance (*p* = 0.153). The non‐WNT/non‐SHH subgroup included the largest proportion of T3b/T4 tumors, suggesting a tendency toward more advanced local disease, whereas WNT tumors showed no T4 lesions.

Surgical and postoperative radiologic variables also differed across groups, although without statistically significant associations. Gross total resection (S1) was achieved more frequently in the WNT subgroup (4/7, 57.1%) than in the SHH (3/10, 30.0%) or non‐WNT/non‐SHH (7/23, 30.4%) groups (*p* = 0.139). Similarly, postoperative MRI‐confirmed absence of residual tumor (R1) was most frequent in WNT tumors, whereas visible residual tumor (R3) predominated in the SHH and non‐WNT/non‐SHH subgroups (*p* = 0.082).

Overall, these findings indicate that the integrated molecular subgroups differed not only in histologic, immunophenotypic, and genomic characteristics but also in their clinical presentation, with WNT tumors tending to occur in older children and showing a more favorable baseline profile, while non‐WNT/non‐SHH tumors were more often associated with metastatic disease and more advanced local extension.

### 3.5 Genomic landscape across integrated molecular subgroups

#### 3.5.1 Recurrent genetic alterations

Distinct subgroup‐associated genetic alteration patterns were identified across the integrated molecular categories, with the most canonical profiles observed in the WNT and SHH subgroups and greater heterogeneity in the non‐WNT/non‐SHH group (Table 2).

**TABLE 2:** Clinically and biologically relevant sequence variants identified across integrated molecular subgroups.

Case ID	Subgroup	Gene	Variant	Protein change	VAF	ACMG	AMP tier	Oncogenicity
MB4	WNT	*CTNNB1*	c.110C>T	p.Ser37Phe	0.44	P	Tier 1	6 (LO)
MB36		*CTNNB1*	c.94G>T	p.Asp32Tyr	0.46	LP	Tier 1	6 (LO)
MB36		*TP53*	c.425C>T	p.Pro142Leu	0.49	LP	Tier 1	4 (MOS)
MB6		*CTNNB1*	c.100G>A	p.Gly34Arg	0.57	P	Tier 1	6 (LO)
MB6		*DDX3X*	c.1051C>T	p.Arg351Trp	0.43	LP	Tier 3	3 (LOS)
MB21		*APC*	c.3183_3187del	p.Gln1062*	0.94	P	Tier 1	5 (MOS)
MB21		*SMARCA4*	c.3490A>C	p.Asn1164His	0.33	VUS	Tier 3	2 (LOS)
MB33		*SMARCA4*	c.3628G>A	p.Val1210Met	0.44	VUS	Tier 3	3 (LOS)
MB5		*SMARCA4*	c.2729C>T	p.Thr910Met	0.55	LP	Tier 2	6 (LO)
MB42		*KMT2D*	c.10819C>T	p.Gln3607*	0.49	P	Tier 2	9 (LO)
MB7	SHH	*PTCH1*	c.2705_2709del	p.Leu902*	0.86	P	Tier 1	9 (LO)
MB7		*ELP1*	c.2822_2826del	p.Lys941Ilefs*2	0.82	LP	Tier 3	1 (U)
MB11		*PTCH1*	c.3158_3168 + 17del	Exon / Intron	0.84	LP	Tier 1	9 (LO)
MB27		*SUFU*	c.182 + 1dup	Splice‐site	1.00	P	Tier 3	9 (LO)
MB27		*KMT2D*	c.7907C>G	p.Ser2636*	0.38	LP	Tier 2	9 (LO)
MB25		*SUFU*	c.37_53del	p.Thr13Trpfs*29	0.95	P	Tier 3	9 (LO)
MB18		*TP53*	c.569C>T	p.Pro190Leu	0.97	LP	Tier 1	6 (LO)
MB8		*TP53*	c.542G>A	p.Arg181His	0.27	P	Tier 1	7 (LO)
MB20		*TERT*	c.‐124C>T	Promoter	0.55	P	Tier 1	8 (LO)
MB15		*TERT*	c.‐124C>T	Promoter	0.37	P	Tier 1	8 (LO)
MB39		*SON*	c.6754A>T	p.Arg2252*	0.28	LP	Tier 3	1 (U)
MB3	Non‐WNT /non‐SHH	*MBD4*	c.939dup	p.Glu314Argfs*13	0.18	P	Tier 3	1 (U)
MB12		*MBD4*	c.939dup	p.Glu314Argfs*13	0.19	P	Tier 3	1 (U)
MB22		*MBD4*	c.939dup	p.Glu314Argfs*13	0.26	P	Tier 3	1 (U)
MB35		*CHEK2*	c.283C>T	p.Arg95*	0.39	P	Tier 1	9 (LO)
MB14		*SON*	c.6754A>T	p.Arg2252*	0.17	LP	Tier 3	1 (U)
MB10		*SON*	c.6754A>T	p.Arg2252*	0.46	LP	Tier 3	1 (U)
MB32		*PMS2*	c.89A>C	p.Gln30Pro	0.22	VUS	Tier 3	2 (LOS)
MB2		*EPHB2*	c.2260G>A	p.Val754Ile	0.57	VUS	Tier 3	1 (U)

Abbreviations: ACMG, American College of Medical Genetics and Genomics; AMP, Association for Molecular Pathology; LO, likely oncogenic; LOS, likely oncogenic support; LP, likely pathogenic; MOS, moderate oncogenic support; P, pathogenic; U, uncertain significance; VAF, variant allele frequency; VUS, variant of uncertain significance.

In the WNT subgroup, recurrent alterations were dominated by genes involved in Wingless signaling, particularly *CTNNB1* and *APC*. Pathogenic or likely pathogenic CTNNB1 variants were detected in 3/7 tumors (MB4, MB6, and MB36), affecting known hotspot residues within the β‐catenin regulatory region, while one additional case (MB21) harbored a truncating *APC* variant. Together, these findings indicate that 4/7 WNT tumors carried canonical WNT‐pathway alterations. Beyond these core subgroup‐defining events, additional co‐occurring variants were identified in genes related to chromatin modulation, RNA metabolism, and genome maintenance, including *SMARCA4* in 3 cases (MB5, MB21, and MB33), *KMT2D* in 1 case (MB42), *DDX3X* in 1 case (MB6), and TP53 in 1 case (MB36). Overall, the WNT subgroup retained a strongly pathway‐focused mutational profile centered on canonical WNT activation, with less frequent secondary events affecting chromatin‐remodeling and transcription‐associated processes.

In the SHH subgroup, the recurrent alterations were centered on genes involved in SHH signaling. Pathogenic or likely pathogenic *PTCH1* alterations were identified in 2/10 tumors (MB7 and MB11), while *SUFU* alterations were detected in 2 additional cases (MB25 and MB27), indicating canonical SHH‐pathway lesions in 4/10 tumors. Recurrent secondary alterations involved genes associated with genome maintenance and RNA metabolism, including *TP53* mutations in 2 cases (MB8 and MB18) and *TERT* promoter alterations in 2 cases (MB15 and MB20). Less frequent events included *ELP1* (MB7), *KMT2D* (MB27), and *SON* (MB39). In addition, *MYCN* amplification was detected in three SHH tumors (MB7, MB8, and MB18), indicating activation of proliferative and transcriptional programs in a subset of cases. Notably, MB18 showed a particularly complex profile, with concurrent *MYCN*, *KRAS*, and *CCND2* amplifications. Taken together, the SHH subgroup demonstrated a genetic landscape anchored by canonical SHH‐pathway activation, with additional recurrent events involving genome maintenance, transcriptional regulation, and chromatin‐associated pathways.

In contrast, the non‐WNT/non‐SHH subgroup displayed the greatest diversity of genetic alterations and lacked a single dominant subgroup‐defining driver. Recurrent alterations in this group most commonly involved genes associated with genome maintenance and RNA metabolism, including MBD4 in 3/23 tumors (MB3, MB12, and MB22) and *SON* in two cases (MB10 and MB14). Additional isolated variants were identified in *CHEK2* (MB35), *PMS2* (MB32), and *EPHB2* (MB2), implicating DNA damage response, mismatch repair, and cell signaling pathways, respectively. In addition, *MYCN* amplification was observed in one non‐WNT/non‐SHH tumor (MB10), indicating that focal transcriptional activation events were not entirely restricted to SHH tumors, although they were distinctly less frequent in this subgroup. Overall, non‐WNT/non‐SHH tumors exhibited a broader and less convergent genetic architecture, with alterations dispersed across genome maintenance, RNA‐processing, and signaling‐related pathways.

Collectively, these findings demonstrate clear subgroup‐associated genetic patterns. The WNT subgroup showed the most canonical and pathway‐focused profile, the SHH subgroup was characterized by recurrent alterations in both SHH signaling and genome maintenance together with focal amplification events in selected cases, and the non‐WNT/non‐SHH subgroup exhibited the greatest heterogeneity, without a unifying sequence‐level driver but with recurrent lesions affecting genome integrity and RNA‐related processes.

#### 3.5.2 Copy‐number alteration patterns

Copy‐number analysis further supported the integrated subgroup classification. In the WNT subgroup, chromosome 6 loss was observed in 6/7 tumors, representing the most characteristic cytogenetic event in this category. Only a limited number of additional copy‐number changes were identified, including isolated 1q gain and 16q loss, consistent with a relatively simple overall CNA profile.

In the SHH subgroup, recurrent CNAs included 9q loss (5 cases), 1q gain (3 cases), 10q loss (3 cases), and 9p gain (2 cases). In addition, four SHH tumors showed isochromosome 17q (i17q). Thus, compared with WNT tumors, the SHH subgroup displayed a broader but still partially recurrent spectrum of copy‐number changes.

The non‐WNT/non‐SHH subgroup exhibited the most complex and heterogeneous copy‐number landscape. The most frequent event was i17q, present in 13/23 tumors, followed by chromosome 8 loss (8/23), chromosome 7 gain (7/23), chromosome 11 loss (5/23), and chromosome 10 loss (5/23). Additional recurrent abnormalities included 1q gain (4/23) and X‐chromosome loss (4/23), together with more scattered imbalances affecting multiple chromosome arms. Overall, these findings indicate that non‐WNT/non‐SHH tumors had the most structurally complex CNA profile among the three subgroups.

Taken together, the integrated approach showed the highest internal concordance in the WNT subgroup, characterized by classic histology, uniform β‐catenin positivity, and recurrent WNT‐associated genomic events, frequently accompanied by chromosome 6 loss. The SHH subgroup also showed substantial concordance, particularly through the combination of desmoplastic/nodular morphology, YAP1 expression, and pathogenic alterations in PTCH1 or SUFU, although some tumors displayed LCA histology or lacked GAB1 positivity. In contrast, the non‐WNT/non‐SHH subgroup was the most heterogeneous, lacking canonical WNT or SHH signatures and showing broader variability in both histologic and genomic findings.

### 3.6 Survival and outcome analysis

Survival outcomes differed across the integrated molecular subgroups. At last follow‐up, all patients in the WNT subgroup were alive (7/7, 100%), whereas deaths were observed in both the SHH and non‐WNT/non‐SHH groups. In the SHH subgroup, 3/10 patients (30.0%) had died and 7/10 (70.0%) remained alive, while the least favorable vital‐status distribution was observed in the non‐WNT/non‐SHH subgroup, in which 11/23 patients (47.8%) had died. Although this comparison did not reach conventional statistical significance, it showed a trend toward differential outcome across molecular groups (*p* = 0.063).

All patients included in the survival analysis were treated according to the same nationally approved pediatric medulloblastoma treatment protocol, with treatment intensity adapted according to predefined clinical risk factors as described in Section 2.2.

**FIGURE 4:**
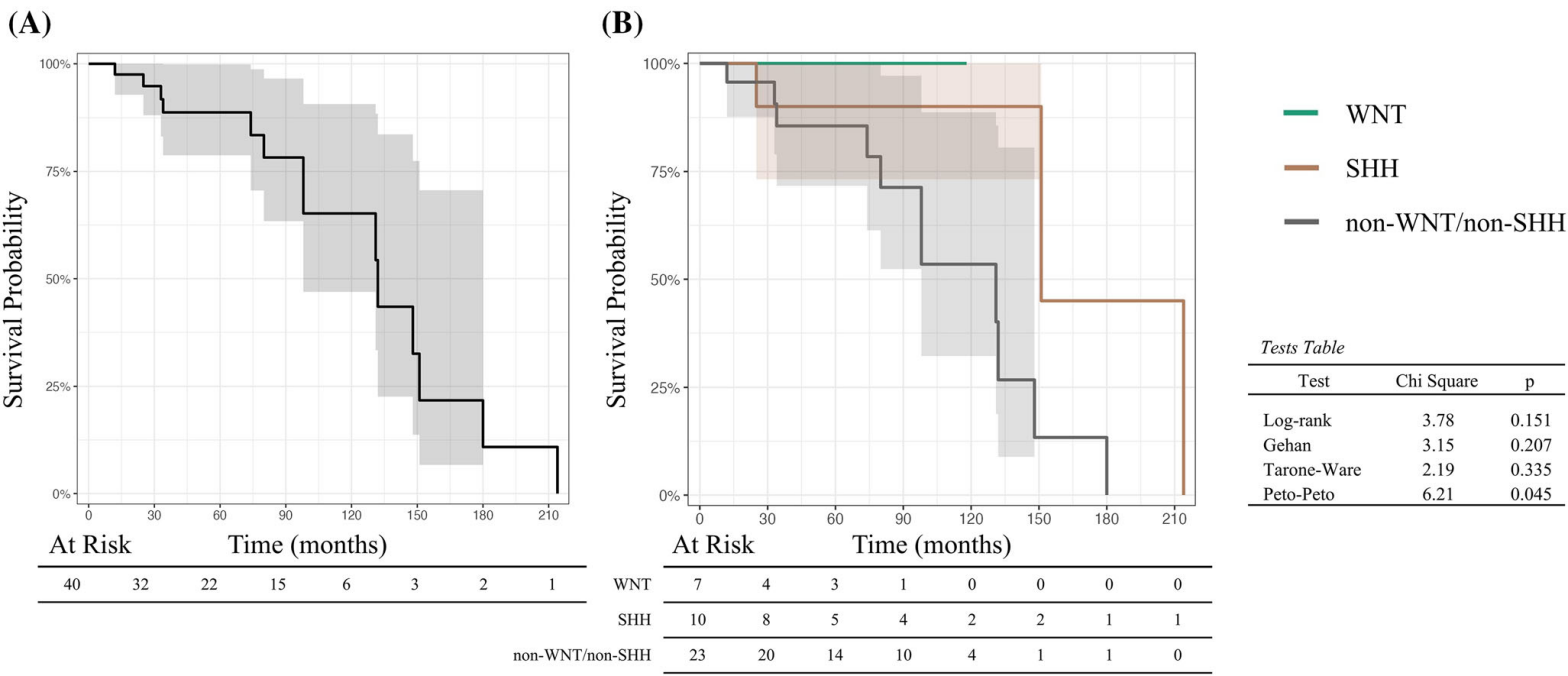
Survival analyses of the pediatric medulloblastoma cohort. (A) Kaplan–Meier overall survival curve for the entire study cohort. Shaded areas represent the 95% confidence intervals, and the table below the plot shows the number of patients at risk at each time point. (B) Kaplan–Meier overall survival curves stratified by integrated molecular subgroup (WNT, SHH, and non‐WNT/non‐SHH). Shaded areas represent the 95% confidence intervals, and the table below the plot shows the number of patients at risk for each subgroup over time. Statistical comparisons between survival curves were performed using the log‐rank, Gehan, Tarone‐Ware, and Peto‐Peto tests.

For the cohort as a whole, Kaplan–Meier analysis demonstrated a gradual decline in OS over time (Figure 4A). The estimated median OS for the entire cohort was 132.0 months, while the 3‐ and 5‐year OS rates were both 88.7% (Table 3). This pattern reflected the relatively limited number of early events, with a greater proportion of deaths occurring later during follow‐up.

**TABLE 3:** Overall survival summary according to integrated molecular subgroup.

Molecular subgroup	*n*	Death, *n* (%)	Median follow‐up (months)	Median OS (months)	3‐year OS, %	5‐year OS (%)
WNT	7	0	45.0	‐	100.0	100.0
SHH	10	3 (30.0)	54.5	151.0	90.0	90.0
Non‐WNT/non‐SHH	23	11 (47.8)	74.0	131.0	85.6	85.6
Overall cohort	40	14 (35.0)	63.0	132.0	88.7	88.7

Abbreviation: OS, overall survival.

Kaplan–Meier analysis stratified by integrated molecular subgroup suggested differences in survival patterns across the three molecular groups (Figure 4B, Table 3); however, these findings should be interpreted cautiously because of the limited number of events within individual subgroups. The WNT subgroup showed the most favorable survival profile, with no recorded deaths during the observation period; accordingly, median OS was not reached, and both the 3‐ and 5‐year OS estimates were 100.0%. The SHH subgroup showed an intermediate survival pattern, with 3 deaths among 10 patients, an estimated median OS of 151.0 months, and 3‐ and 5‐year OS rates of 90.0%. These survival estimates represent the overall outcome of the entire SHH cohort and should not be interpreted as reflecting the prognosis of TP53‐defined SHH subgroups. Because only two SHH tumors harbored TP53 alterations, no meaningful survival comparison between SHH‐TP53‐mutant and SHH‐TP53‐wildtype tumors could be performed, precluding subgroup‐specific analyses according to TP53 status. Specifically, one TP53‐mutant SHH patient remained alive after 104 months of follow‐up, whereas the second survived 151 months after diagnosis before death, illustrating the marked clinical heterogeneity within this subgroup. In contrast, the non‐WNT/non‐SHH subgroup had the least favorable long‐term survival, with 11 deaths among 23 patients, an estimated median OS of 131.0 months, and 3‐ and 5‐year OS rates of 85.6%.

Despite these apparent differences in survival trajectories, the global log‐rank test did not demonstrate a statistically significant difference among the three groups (χ^2^ = 3.78, *p* = 0.151). Exploratory weighted comparisons showed partial sensitivity to differences emerging later during follow‐up, with the Peto‐Peto test reaching statistical significance (χ^2^ = 6.21, *p* = 0.045), whereas the Gehan and Tarone‐Ware tests remained non‐significant (*p* = 0.207 and *p* = 0.335, respectively). Given the limited sample size and number of events, these exploratory findings should be interpreted cautiously and considered hypothesis‐generating rather than confirmatory.

Interpretation of subgroup‐specific survival estimates should take into account the timing of events. In both the SHH and non‐WNT/non‐SHH subgroups, several deaths occurred beyond 60 months, which explains why the 5‐year OS estimates remained relatively high despite less favorable long‐term survival. Overall, the observed survival patterns were consistent with the integrated molecular classification and were evaluated within a relatively uniform national treatment framework, with the most favorable outcome observed in WNT tumors, intermediate outcome in SHH tumors, and the least favorable survival profile in the non‐WNT/non‐SHH subgroup. Nevertheless, these findings should be interpreted as exploratory because of the limited cohort size and the relatively small number of outcome events within individual molecular subgroups.

## 4 DISCUSSION

To our knowledge, this is the first study to characterize the molecular landscape of pediatric medulloblastoma in Kazakhstan using an integrated FFPE‐applicable workflow combining histopathology, immunohistochemistry, WES, and copy‐number analysis. Despite the absence of DNA methylation profiling, all tumors could be assigned to clinically meaningful molecular categories using an integrated diagnostic framework. The observed subgroup distribution, clinicopathologic characteristics, genomic alterations, and survival patterns were broadly consistent with the established biology of pediatric medulloblastoma, supporting the feasibility of this approach in routine diagnostic practice where comprehensive molecular platforms are not readily available.

### 4.1 Comparison with previous studies

The molecular distribution observed in our cohort closely resembles that reported in large international pediatric series, with non‐WNT/non‐SHH tumors representing the largest subgroup and WNT tumors the least frequent. Likewise, the age distribution of the molecular groups was consistent with previous studies, with WNT tumors occurring predominantly in older children, SHH tumors spanning a wider age range including infants, and non‐WNT/non‐SHH tumors clustering mainly during childhood [5, 19, 42].

Histologic, immunophenotypic, and genomic findings also paralleled the established biology of medulloblastoma. WNT tumors demonstrated the expected combination of classic morphology, nuclear β‐catenin accumulation, recurrent *CTNNB1*/*APC* alterations, and chromosome 6 loss, whereas SHH tumors were enriched for desmoplastic/nodular histology, YAP1 expression, and pathogenic alterations involving *PTCH1* or *SUFU*. In contrast, non‐WNT/non‐SHH tumors showed the greatest histologic and genomic heterogeneity, including frequent i17q, reflecting the biological diversity of the Group 3/Group 4 continuum [1, 2, 3, 4, 5, 6, 7, 17, 40]. Collectively, these findings support the validity of the integrated classification strategy despite the absence of methylation‐based subclassification.

An important observation was that canonical pathway‐defining genomic alterations were not identified in every molecularly assigned WNT or SHH tumor. Pathogenic *CTNNB1*/*APC* alterations were detected in 4/7 WNT tumors (57.1%), whereas pathogenic *PTCH1*/*SUFU* alterations were identified in 4/10 SHH tumors (40.0%). Previous genomic studies have similarly demonstrated that these canonical alterations are characteristic but not universal. WNT tumors lacking *CTNNB1* mutations may instead harbor *APC* alterations or other mechanisms of WNT‐pathway activation, while SHH tumors exhibit considerable genomic heterogeneity involving *SMO*, *GLI2*, *MYCN*, structural rearrangements, CNAs, and epigenetic mechanisms that are not necessarily captured by WES [5, 43, 44]. These observations reinforce current WHO recommendations that molecular subgroup assignment should rely on integrated interpretation of morphologic, immunophenotypic, and genomic findings rather than any single molecular alteration.

Although survival analyses were limited by the modest cohort size, the overall prognostic gradient remained consistent with previous reports, with the most favorable outcomes observed in WNT tumors and less favorable long‐term survival among non‐WNT/non‐SHH tumors [8, 45, 46, 47, 48]. The absence of methylation‐based discrimination between Group 3 and Group 4 likely reduced the ability to detect statistically significant survival differences, as these biologically distinct entities were necessarily analyzed together.

### 4.2 Diagnostic implications for resource‐limited settings

One of the principal strengths of this study is the demonstration that clinically informative molecular stratification can be achieved using routinely available FFPE material through integration of histopathology, immunohistochemistry, WES, and copy‐number analysis. This approach reflects the practical reality of many diagnostic laboratories where DNA methylation profiling remains unavailable because of financial, technical, or infrastructural constraints.

Our findings also illustrate the strengths and limitations of currently available immunohistochemical surrogate markers. Although β‐catenin and YAP1 showed good concordance with the integrated molecular classification, GAB1 expression was detected in only a minority of SHH tumors, indicating that this marker alone lacks sufficient sensitivity for reliable subgroup assignment in our cohort. Similar variability has been reported previously, particularly in archival FFPE material and desmoplastic/nodular tumors, emphasizing that GAB1 should be regarded as a supportive rather than defining marker [5, 49, 50, 51, 52]. Recent studies have proposed expanded immunohistochemical panels incorporating LEF1, filamin A, OTX2, and p75‐NGFR, which may improve subgroup discrimination in laboratories without access to DNA methylation profiling [35, 52]. Nevertheless, no individual immunohistochemical marker provides sufficient diagnostic accuracy in isolation, underscoring the importance of integrated interpretation.

An additional consideration for resource‐adapted diagnostic workflows is the evaluation of *TP53* status in SHH‐activated medulloblastoma. p53 immunohistochemistry provides a rapid and inexpensive screening tool and has demonstrated excellent sensitivity for detecting TP53 alterations, although specificity is lower and both diffuse overexpression and complete loss of staining should be interpreted as abnormal [5, 53, 54]. Accordingly, p53 immunohistochemistry is best regarded as a triage test to prioritize cases for molecular confirmation rather than as a replacement for sequencing.

Interestingly, both *TP53*‐mutant SHH tumors in our cohort demonstrated relatively prolonged survival despite their LCA histology. One patient remained alive after 104 months of follow‐up, whereas the second survived 151 months after diagnosis before death. Although SHH‐*TP53*‐mutant medulloblastomas are generally associated with poor prognosis, these observations should be interpreted cautiously because only two *TP53*‐mutant tumors were identified. Individual treatment response, extent of resection, and other clinicopathologic factors may have contributed to these outcomes, precluding meaningful conclusions regarding the prognostic impact of *TP53* alterations in our cohort [55, 56].

Overall, our findings suggest that an integrated FFPE‐based diagnostic workflow represents a practical bridge between conventional neuropathology and comprehensive molecular classification. Although this strategy does not replace DNA methylation profiling, which remains the reference standard for resolving Group 3, Group 4, and second‐generation molecular subtypes, it enables clinically meaningful identification of canonical WNT and SHH tumors while avoiding overinterpretation of cases that cannot be confidently subclassified. This approach may facilitate broader implementation of molecular diagnostics in resource‐limited settings and contribute to more equitable access to precision neuro‐oncology [14, 16, 57].

### 4.3 Strengths and limitations

This study has several strengths. It provides, to our knowledge, one of the first subgroup‐oriented molecular characterizations of pediatric medulloblastoma from Kazakhstan, addressing a regional evidence gap in an underrepresented setting. The study design combined histology, immunohistochemistry, WES, and copy‐number analysis, allowing a multidimensional assessment of tumor biology rather than reliance on a single diagnostic layer. Another strength is the use of archived FFPE material, which reflects real‐world practice and supports the feasibility of retrospective molecular stratification in settings where fresh‐frozen tissue and advanced reference‐platform testing are not routinely available. In addition, the inclusion of clinicopathologic variables and survival data enabled preliminary evaluation of the clinical relevance of the identified molecular categories.

Several limitations should also be acknowledged. First, the study was based on a retrospective single‐center cohort with a relatively small sample size, which limits statistical power, particularly for subgroup comparisons and survival analyses. Second, DNA methylation profiling and transcriptome‐based classification were not available; therefore, tumors outside the canonical WNT and SHH categories were retained within a broader non‐WNT/non‐SHH group and could not be resolved into Group 3 and Group 4 or into more refined second‐generation subtypes. This limitation reflects the current biological and methodological understanding of medulloblastoma rather than a technical failure of the present study. Although WES successfully identified subgroup‐associated sequence variants and CNAs, current evidence indicates that reliable discrimination between Group 3 and Group 4 requires DNA methylation profiling or transcriptomic approaches, which remain the reference standard for resolving these epigenetically defined entities. Accordingly, tumors lacking canonical WNT or SHH features were intentionally retained within the broader non‐WNT/non‐SHH category to avoid overinterpretation of the available molecular data. Third, the use of FFPE‐derived DNA and a tumor‐only sequencing design may have limited sensitivity for some alterations and constrained discrimination between somatic and germline events in selected cases. Fourth, not all clinicoradiologic variables were available for every patient, and some outcome analyses should therefore be interpreted cautiously.

These limitations notwithstanding, the study demonstrates that an FFPE‐applicable integrated workflow can still provide biologically and clinically informative medulloblastoma stratification in routine practice. The findings should be viewed as a foundation for future larger‐scale studies incorporating multicenter recruitment, methylation‐based classification, transcriptomic characterization where appropriate, and more comprehensive molecular resolution of non‐WNT/non‐SHH tumors.

## 5 CONCLUSION

In conclusion, this study provides one of the first subgroup‐oriented molecular profiles of pediatric medulloblastoma in Kazakhstan using an FFPE‐applicable framework that combines histology, immunohistochemistry, WES, and copy‐number analysis. The cohort showed biologically coherent stratification into WNT, SHH, and non‐WNT/non‐SHH categories, with canonical WNT‐pathway alterations and chromosome 6 loss in WNT tumors, *PTCH1*/*SUFU*‐associated SHH‐pathway lesions and recurrent secondary alterations in SHH tumors, and greater genomic and structural heterogeneity in the non‐WNT/non‐SHH subgroup. The identified molecular categories were also associated with clinically meaningful differences in age distribution, metastatic presentation, and outcome, with the most favorable survival observed in WNT tumors and the least favorable profile in non‐WNT/non‐SHH tumors. Although methylation‐based subclassification was not available, these findings show that pragmatic, FFPE‐based molecular profiling can provide biologically and clinically informative medulloblastoma stratification in routine practice and may serve as a foundation for future larger‐scale, methylation‐informed studies in Kazakhstan and other underrepresented settings.

## AUTHOR CONTRIBUTIONS

Conceptualization: **AB**, **AZ**, and **MB**; methodology: **AB**, **AZ**, and **MB**; validation: **AA**, **MS**, **BI**, **ZZ**, and **BZ**; formal analysis: **AB** and **XX**; investigation: **AZ**, **AA**, **MS**, **BI**, **ZZ**, **GÓ**, and **BZ**; data curation: **AZ**, **AA**, **MS**, **BI**, **ZZ**, **GÓ**, and **BZ**; writing—original draft preparation: **AB**; writing—review and editing: **AZ**, **AA**, **MS**, **BI**, **ZZ**, **GÓ**, **BZ**, **XX**, and **MB**; visualization: **AB** and **ZZ**; supervision: **MB**; project administration: **MB**; funding acquisition: **MB**. All authors have read and agreed to the published version of the manuscript.

## FUNDING INFORMATION

This research has been funded by the Committee of Science of the Ministry of Science and Higher Education of the Republic of Kazakhstan (grant title: Study of molecular genetic features and monitoring of the effectiveness of medulloblastoma therapy in children: a step toward personalized therapy, grant No. AP23490016).

## CONFLICT OF INTEREST STATEMENT

The authors declare no conflicts of interest.

## ETHICS STATEMENT

This study was approved by the Local Committee of Bioethics of the “University Medical Center” Corporate Fund, Kazakhstan (Protocol number: №8/2024/ПЭ, date: 03.09.2024).

## CONSENT

Written informed consent for participation and for the use of clinical records and biomaterial in anonymized research was obtained from the legal representatives of patients whenever feasible during the study period. For archived cases, the original hospitalization consent documentation included permission for the use of anonymized clinical data and biological material for research purposes.

## Data Availability

The raw genomic data supporting the findings of this study are not publicly available because of concerns regarding genetic data privacy and the sensitive nature of genomic information, particularly involving children. Instead, the data can be accessed upon reasonable request from the corresponding author.
